# Enhancing Magnetic Resonance Imaging (MRI) Report Comprehension in Spinal Trauma: Readability Analysis of AI-Generated Explanations for Thoracolumbar Fractures

**DOI:** 10.2196/69654

**Published:** 2025-07-01

**Authors:** David C Sing, Kishan S Shah, Michael Pompliano, Paul H Yi, Calogero Velluto, Ali Bagheri, Robert K Eastlack, Stephen R Stephan, Gregory M Mundis Jr

**Affiliations:** 1 Division of Spine Surgery, Department of Orthopaedic Surgery, Scripps Clinic, 10710 N Torrey Pines Rd, La Jolla, CA, 92037, United States, 1 8585547988; 2 Department of Radiology, St. Jude Children's Research Hospital, Memphis, TN, United States

**Keywords:** ChatGPT, AI, artificial intelligence, LLM, large language model, patient education, orthopedic surgery, MRI, magnetic resonance imaging, thoracolumbar fracture, spine surgery, trauma

## Abstract

**Background:**

Magnetic resonance imaging (MRI) reports are challenging for patients to interpret and may subject patients to unnecessary anxiety. The advent of advanced artificial intelligence (AI) large language models (LLMs), such as GPT-4o, hold promise for translating complex medical information into layman terms.

**Objective:**

This paper aims to evaluate the accuracy, helpfulness, and readability of GPT-4o in explaining MRI reports of patients with thoracolumbar fractures.

**Methods:**

MRI reports of 20 patients presenting with thoracic or lumbar vertebral body fractures were obtained. GPT-4o was prompted to explain the MRI report in layman’s terms. The generated explanations were then presented to 7 board-certified spine surgeons for evaluation on the reports’ helpfulness and accuracy. The MRI report text and GPT-4o explanations were then analyzed to grade the readability of the texts using the Flesch Readability Ease Score (FRES) and Flesch-Kincaid Grade Level (FKGL) Scale.

**Results:**

The layman explanations provided by GPT-4o were found to be helpful by all surgeons in 17 cases, with 6 of 7 surgeons finding the information helpful in the remaining 3 cases. ChatGPT-generated layman reports were rated as “accurate” by all 7 surgeons in 11/20 cases (55%). In an additional 5/20 cases (25%), 6 out of 7 surgeons agreed on their accuracy. In the remaining 4/20 cases (20%), accuracy ratings varied, with 4 or 5 surgeons considering them accurate. Review of surgeon feedback on inaccuracies revealed that the radiology reports were often insufficiently detailed. The mean FRES score of the MRI reports was significantly lower than the GPT-4o explanations (32.15, SD 15.89 vs 53.9, SD 7.86; *P*<.001). The mean FKGL score of the MRI reports trended higher compared to the GPT-4o explanations (11th-12th grade vs 10th-11th grade level; *P*=.11).

**Conclusions:**

Overall helpfulness and readability ratings for AI-generated summaries of MRI reports were high, with few inaccuracies recorded. This study demonstrates the potential of GPT-4o to serve as a valuable tool for enhancing patient comprehension of MRI report findings.

## Introduction

The 21st Century Cures Act has recently mandated that medical imaging exam results be made immediately available to patients after the radiologist report is finalized [1]. In many situations, patients will review their imaging reports without guidance from a medical professional, leading to confusion and anxiety [2]. Ideally, an ordering physician would be able to review the imaging results with their patients in a timely fashion, but this is often not the case. There is, therefore, a need for patients to be able to more easily and efficiently interpret the text of their imaging reports.

As large language models (LLMs) rapidly become more sophisticated and powerful, the premise of using artificial intelligence (AI)–generated summaries of imaging reports as a tool that may assist clinicians in improving communication with patients has been gaining support. Recent analyses of ChatGPT-4o (OpenAI) have demonstrated its effectiveness and accuracy in summarizing diagnostic radiology reports, with the ability to translate medical terminology to an 8th-grade reading level [3]. When prompted to explain medical imaging reports to a child using simplified and basic language, ChatGPT-4o generated 15 different reports, which were evaluated by 15 radiologists. The overall consensus was that the reports were factually correct, complete, and did not pose any harm for misinformation [4].

Management of thoracolumbar fractures is a particularly challenging aspect of patient care for spine surgeons. Regional variation in treatment approach methodology contributes to a larger widespread inconsistency in the standard of care [5,6]. Therefore, management of vertebral body fractures is heavily influenced by an individual surgeon’s experience and comfort level. The heterogeneity of spinal fracture morphology also results in more complex terminology in magnetic resonance imaging (MRI) reports.

Recent analyses have demonstrated that high quality educational content is available for patients, offering appropriate counseling on osteoporosis and bone health, diagnosing and treating cervical radiculopathy, as well as answering commonly asked questions about spinal cord injury [7-9]. However, thus far, no reports have been published on the accuracy and helpfulness of ChatGPT-generated explanations of spinal trauma MRI reports, specifically thoracolumbar fractures, in the emergency department. Therefore, the objective of our study was to evaluate the readability of ChatGPT-generated layperson summaries of radiology reports for 20 patient cases of thoracolumbar fractures. We hypothesized that ChatGPT-generated summaries would help provide clearer and more understandable MRI report findings that contain accurate explanations of imaging findings without any “hallucinated” or fabricated content—a flaw observed in earlier LLM versions where the AI program would often invent facts or cite nonexistent literature without clearly acknowledging the fabricated content.

## Methods

### Study Design

**Figure 1.**
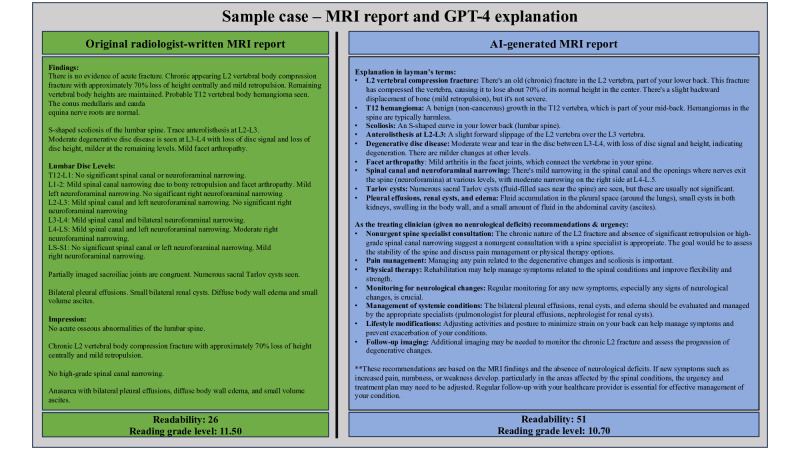
This diagram showcases how deidentified magnetic resonance imaging (MRI) reports were processed through ChatGPT-4o with a prompt that asked to explain the imaging findings in layman’s terms for patient education purposes. The MRI report completed by the radiologist can be seen on the left in green, while a GPT-4o–generated, simplified version of the MRI report can be seen on the right in blue. Readability scores (Flesch Readability Ease Score) and reading grade levels (Flesch-Kincaid Grade Level) were determined for each version of the MRI report. AI: artificial intelligence.

Searching our institutional Picture Archiving and Communications System (PACS), we identified 20 patients who presented to the emergency department at a level 1 trauma center and underwent MRI for evaluation of an acute thoracolumbar vertebral body fracture. Each patient was evaluated urgently through consultation with 1 of 7 board-certified spine surgeons providing on-call coverage. These 20 consecutive encounters all occurred between 2023 and 2024. A total of 20 patient cases were chosen in order to sufficiently include a variety of different clinical scenarios with varying types of fracture morphology and severity. MRI was chosen in favor of other imaging modalities as MRI reports are generally more challenging to interpret, as they often contain varying complex descriptors of combined ligamentous and bony injuries, making each case unique and nuanced. Reports were deidentified by excluding the patient’s name, date of study, and radiologist’s name from the reports. These deidentified reports were then submitted to ChatGPT-4o with the prompt, “Explain in layman terms with as much detail as possible.” This prompt was selected over others, as it concise, yet specific with regards to the desired output as a patient education tool (see Figure 1).

The GPT-4o–generated layman summaries of the MRI reports were formatted into an electronic survey for evaluation by the same 7 on-call board-certified spine surgeons at the level 1 trauma center emergency department. The surgeons were asked to grade each prompt as “helpful” or “not helpful” and “accurate” or “not accurate.” Descriptive statistics were used to summarize surgeon ratings of helpfulness and accuracy, with results reported as frequencies and percentages.

### Statistical Analysis

Readability of the original MRI report written by the radiologist, as well as the GPT-4o layman report, was analyzed using an internet-based readability scoring system [10]. The first measure of readability calculated was the Flesch Reading Ease Score (FRES; 1 to 100, with 100 being the highest readability score; see Equation 1). The second measure of readability assessed was the Flesch-Kincaid Grade Level (FKGL) scale (approximating the reading grade level of a text; see Equation 2). Readability scores were then statistically analyzed using a paired *t* test to compare readability scores between the original MRI reports written by the radiologist and GPT-4o–generated layman explanations in order to assess whether there was a significant difference in FRES and FKGL scores. Paired *t* tests were performed on the exact FKGL and FKRE values (not ranges) to assess statistical significance. A *P* value of<.05 was considered statistically significant.


(1) $$206.835-1.015\left(\frac{total\;words}{total\;sentences}\right)-84.6\left(\frac{total\;syllables}{total\;words}\right)$$

(2) $$0.39\left(\frac{total\;words}{total\;sentences}\right)+11.8\left(\frac{total\;syllables}{total\;words}\right)-15.59$$

In addition, interrater reliability among the 7 surgeons evaluating MRI reports was assessed using Cohen kappa statistic in an effort to quantify agreement beyond chance.

### Ethical Considerations

The Scripps Health Institutional Review Board approved this study with a waiver for deidentified use of patient records. This study was conducted in accordance with the ethical standards of the Declaration of Helsinki and was approved by the Department of Orthopaedic Surgery at Scripps Health and the San Diego Spine Foundation.

## Results

### Surgeon Evaluation of Helpfulness and Accuracy

A total of 20 noncontrast MRI reports of the lumbar spine were included in this study. In total, 17 of the 20 layman reports (85%) were unanimously determined to be “helpful” by all 7 surgeons, while the remaining 3 reports were considered “helpful” by 6 of the 7 surgeons (see Table 1). In terms of accuracy, surgeons unanimously rated 11 of the 20 layman reports (55%) as “accurate.” An additional 5 reports (25%) were rated as “accurate” by 6 of 7 surgeons, while the remaining 4 reports (20%) received mixed ratings, with 4 or 5 surgeons agreeing on their accuracy. Notably, however, at least half of all surgeons surveyed rated every layman MRI report as “accurate.” In the 4 cases where only 2 or 3 surgeons rated the layman reports as “inaccurate,” the original radiology reports lacked sufficient detail (see Table 2). In these instances, surgeons indicated they would prefer to personally review the imaging studies before determining the accuracy of the explanations. Interrater reliability was high among surgeons (κ=0.80), as well as between surgeon consensus and GPT-4o (κ=0.90).

**Table 1.** Surgeon ratings of helpfulness for GPT-4o layman reports.

Helpfulness rating	Reports, n	Surgeon agreement, n/N	Percentage
Unanimously helpful	17	7/7	85
Majority considered helpful	3	6/7	15

**Table 2.** Surgeon ratings of accuracy for GPT-4o layman reports.

Accuracy rating	Cases (N=20), n (%)
All 7 surgeons agreed (accurate)	11 (55)
6 of 7 surgeons agreed (accurate)	5 (25)
4 or 5 of 7 surgeons agreed (mixed ratings)	4 (20)

### FKGL and FRES Readability Analysis

The readability of the MRI reports and their GPT-4-generated layman explanations was evaluated using the FRES and FKGL metrics.

The MRI reports had FRES scores ranging from 7 to 61, with a mean of 32.15 (SD 15.89), indicating that the text was classified as “difficult to read” by standard readability metrics (see Table 3). In contrast, the GPT-4 explanations had FRES scores ranging from 40 to 72, with a mean of 53.9 (SD 7.86), demonstrating a substantial improvement in readability. The average increase in FRES score between the original MRI report and GPT-4o report was +21.75 points, which was statistically significant (*P*<.001). This result confirms that GPT-4 effectively enhanced the readability of MRI report findings, making them considerably easier for patients to understand.

**Table 3.** Reading ease and reading grade scoring comparing magnetic resonance imaging (MRI) reports and GPT-4o explanations.

Case	FRES^a^ score			FKGL^b^ score		
	Original MRI report	GPT-4o report	Difference	Original MRI report	GPT-4o report	Difference
Case 1	29	55	26	11.63	10.58	−1.05
Case 2	19	72	53	13.61	7.62	−5.99
Case 3	15	56	41	13.72	11.26	−2.46
Case 4	59	68	9	8.43	8.93	0.5
Case 5	36	52	16	11.42	11.44	0.02
Case 6	39	56	17	9.48	10	0.52
Case 7	24	57	33	13.19	9.44	−3.75
Case 8	7	55	48	14.76	9.97	−4.79
Case 9	26	59	33	12.55	9.61	−2.94
Case 10	61	58	−3	9.02	10.8	1.78
Case 11	45	40	−5	8.90	12.2	3.3
Case 12	8	40	32	15.55	13.02	−2.53
Case 13	46	46	0	10.54	12.56	2.02
Case 14	20	53	33	13.32	11.44	−1.88
Case 15	46	56	10	9.67	10.36	0.69
Case 16	32	46	14	11.28	10.77	−0.51
Case 17	20	45	25	13.5	11.95	−1.55
Case 18	26	53	27	10.92	10.70	−0.22
Case 19	59	60	1	8.43	10.16	1.73
Case 20	26	51	25	11.45	10.69	−0.76

a FRES: Flesch Reading Ease Score; scale of 1 to 100, with 100 being the highest readability score.

b FKGL: Flesch-Kincaid Grade Level; assess the approximate reading grade level of a text.

The original MRI reports had an FKGL score ranging from 8.43 to 15.55, with a mean of 11.57 (SD 2.10), indicating that a high school to early college-level reading proficiency was required for full comprehension (see Table 3). In comparison, the GPT-4o–generated explanations had FKGL scores ranging from 7.62 to 13.02, with a mean of 10.67 (SD 1.24), representing a reduction in the required reading level for full comprehension. On average, the GPT-4o summaries lowered the FKGL score by 0.89 grade levels; however, this reduction did not reach statistical significance (*P*=.11). This suggests that while GPT-4o was effective in simplifying the reports as seen by the significant improvement in FKRE scores, some medical complexity still remained, as seen by the nonsignificant improvement in FKGL scores. This could still pose comprehension challenges for patients with lower health literacy.

### Incidental Findings

8 out of 20 MRI reports (40%) reported incidental findings unrelated to spinal trauma in the MRI report. These findings included hemangiomas, renal cysts, thick-walled esophagus, epidural lipomatosis, bile duct ectasia, perineural root sleeve cysts, dorsal epidural lipomatosis, and Tarlov cysts. These incidental findings were all appropriately comprehended by LLM and explained in the GPT-4o–generated report to be likely benign, with recommendation for monitoring with follow-up.

## Discussion

### Principal Findings

This study demonstrates that AI, specifically GPT-4o, has the immense potential to produce accurate and helpful explanations that improve patient comprehension of MRI report findings. All 7 board-certified spine surgeons surveyed in this study reached consensus that the tool was both useful and lacked any major inaccuracies. Only 3 GPT-4o–generated reports contained potential inaccuracies, but this was determined to be due to a lack of detail in the original radiologist-written report. Furthermore, incidental findings that often cause anxiety, including common benign tumors such as hemangiomas or renal cysts, were accurately explained by GPT-4o to be unrelated to the present injury and likely benign, with recommendation for appropriate follow-up.

The improvements seen in FRES scores suggest that GPT-4o–generated explanations significantly enhance text clarity and patient accessibility. The lack of statistical significance in FKGL score reduction suggests that while GPT-4o lowers the reading grade level, some complex medical terminology and sentence structure remains–addressing this gap will require further refinement of LLMs for optimal patient comprehension. Given that MRI reports are often written at a high school or college reading level, the ability of GPT-4o to improve readability while maintaining accuracy is particularly relevant for patient education. Patients with lower health literacy may benefit from structured AI-generated summaries, potentially reducing anxiety and misunderstandings regarding their diagnosis. However, given the residual complexity in some explanations provided by GPT-4o, integrating human oversight in AI-assisted patient education remains crucial until further improvement in LLMs is seen in the future.

### Addressing the Communication Gap in Medical Imaging

While medical imaging is often relied upon significantly in the decision-making process for cases that may require surgery, the complexity of the reports, which are now mandated to be made immediately available to the patient, can cause undue stress to patients who lack the means to interpret unfamiliar medical jargon [11,12]. These MRI studies are also commonly ordered by primary care and emergency department providers, who often rely on spine surgeon consultation to educate patients. The value of a resource like GPT-4o lies primarily in bridging the communication gap between spine surgeons, other members of the patient care team, and the patient [13]. For example, surgeons may often be unavailable or delayed when a patient or an emergency department clinician seeks help reviewing a study. Incorporating GPT-4o generated MRI reports, in these situations, can allow for more efficient and precise care, which results in better patient-reported outcomes in the long term. Although further input from surgeons is necessary before formal adoption, it is conceivable that nurses, physician assistants, and emergency department providers may be able to enhance their understanding and interpretation of MRI report findings with the use of GPT-4o–generated reports, allowing them to counsel patients with confidence and prevent them from making detrimental clinical decisions for patients.

### Existing Literature in This Field

While ChatGPT’s use as a patient education tool has been examined in previous studies, limited literature exists on application of LLMs like ChatGPT in summarizing radiology reports [14,15]. Other assessments of ChatGPT in deciphering MRI reports of knee and shoulder injuries demonstrated similar usefulness and relevant explanations [16]. A review of ChatGPT-generated explanations of 20 MRI shoulder, 20 MRI knee, and 20 MRI lumbar spine reports showed high overall ratings for accuracy and completeness, as only 3 explanations out of the 60 reviewed reports were deemed confusing or inaccurate [17]. ChatGPT also performed well in explaining chest CT and brain MRI reports, as Lyu et al. concluded that the AI-generated explanations efficiently and effectively translated complex information into plain language without direct involvement from a human expert [18].

### Limitations

A so-called “hallucination” refers to any AI-generated output that contains completely fabricated content that is both factually incorrect and unrelated to content from the original MRI report written by the Radiologist. In agreement with many other previous studies cited above, our research found no “hallucinations.” This may be due to our use of intentionally crafted, highly specific prompts. Nonetheless, the possibility of an LLM generating inaccurate information is certainly plausible, though it was overwhelmingly rare in this specific use case, with no instances of gross inaccuracy or fabrication found in our study. For this reason, GPT-4o has the potential to be used as a supplementary resource with oversight and contextual judgment by clinicians at this point in time. Other limitations include the diversity of possible end-users who are tasked with interpreting reports. Though the output was reviewed by spine surgeons in this study, Radiologists and emergency department clinicians would also need to feel comfortable with the appropriateness and accuracy of the tool. Further interdisciplinary surveys to examine their assessment of the GPT-4o–generated reports would be valuable in addition to this study. Ultimately, the surgeon, along with any end-users who make clinical decisions based on MRI studies, should oversee the appropriate use of ChatGPT-generated layman explanations. There may be unforeseen risks with regards to providing inadequate clinical care or counseling without surgeon oversight, especially in the emergency department setting where patients may present with life-altering injuries. Further limitations include a limited sample size of 20 cases with a narrow, focused cohort of patients presenting with thoracolumbar fractures only. Counseling for incidental findings often recommended specialist visits; however, these cases would be more appropriately addressed with an initial evaluation by a primary care provider first.

### Expanding Capabilities: Multimodal and Multilingual Applications

Although these limitations affect the current clinical usage of LLMs, ongoing advancements in their development continue to expand their potential in the medical sphere. Recent progress has demonstrated that LLMs can now analyze digital images alongside text, further enhancing their applicability in medical imaging analysis and presentation to patients. This multimodal capacity will enhance the usability of ChatGPT as a patient education tool. In the future, image recognition and analysis capabilities may allow ChatGPT to conduct its own analysis of imaging studies, and complementing or correlating to the radiologist’s report. Significant advances have been demonstrated with foreign language translation, additionally aiding non-English speaking patients with high accuracy, consistency, fluency, and contextual awareness in translating text [19]. This feature would allow GPT-4o and even more advanced version of the model to analyze radiology reports that are currently being outsourced to Radiologists to read in different countries, and provide accurately translated layman reports for patients to read almost instantaneously. As LLMs continue to improve, the translation of medical jargon to layman’s terms will inevitably become increasingly accurate and effective.

### Addressing the Inherent Variability of Radiology Reports

A persistent limitation for AI-generated explanations, specifically in the medical field and radiology space, has been the inherent variability and occasional insufficiency of details in radiology reports [20]. As reported in this study, spine surgeons at times noted the lack of detail in ChatGPT-generated reports and cited this topic as an issue that needs to be addressed. The problem of insufficient data in reports will always be a limiting factor, as more or less detail may be required in certain reports based on the patient’s particular unique presentation. Improving AI accuracy may require mimicking how clinicians approach image review, incorporating both pattern recognition and contextual judgment rather than relying only on textual descriptions written in reports [21]. Developing AI models that align more closely with how doctors synthesize image findings with clinical context could enhance the accuracy and usefulness in real-world applications.

### Future Directions, Considerations, and Implications

A potential next step to enhance accuracy would be fine-tuning or retraining the model on a larger dataset of past radiology reports and corresponding expert-reviewed layperson explanations. This would allow the LLM to recognize complex fracture patterns from a particular report more reliably and improve consistency in terminology use [22]. In addition, integrating contextual memory, whereby the model retains past patient-specific information across reports, could improve continuity and personalization in explanations. Future research should be centered around using specialized medical fine-tuning techniques and human-in-the-loop verification to optimize AI-generated patient education tools [23]. Improvements in AI-generated patient education tools should focus on model retraining with expert annotated datasets to enhance accuracy and consistency. Furthermore, implemented adaptive learning mechanisms–where the AI refines its outputs based on clinician feedback–could further improve the reliability of the reports generated.

From a clinical perspective, a significant predictor of fracture instability is the competency of the posterior ligamentous complex (PLC), which is often not described in radiology reports [24]. Despite GPT-4o having an incomplete description of the fracture pattern, it was still able to describe and make the right interpretation from what was included in Radiologist’s report without making inaccurate assumptions. It is well known that LLMs like ChatGPT have intrinsic biases based on the initial training data used to create the model [25]. Reproducing of the quality or accuracy of the reports in future analyses may be difficult to achieve as prompting the LLM with the same prompt may result in different, but semantically similar responses. Metastatic or infection-related fractures were deemed outside the scope of this study, as interpretation of MRI reports describing less frequently occurring “edge cases” may also require future in-depth analysis. From an ethical standpoint, the sharing deidentified patient information with ChatGPT or other AI programs may also raise ethical concerns that could hinder future improvement and optimization of LLM capabilities.

Despite these challenges, all 7 on-call spine surgeons all acknowledged the significant potential of ChatGPT to enable patient-centered care by providing simplified and more comprehensible explanations of advanced imaging reports. Patients are often discharged from the emergency department without complete understanding on the extent of their injury or whether or not they may need surgery [26]. When patients are instructed to follow-up with a spine surgeon, they often feel stress and anxiety, as the consultation may imply the need for surgical intervention [27]. In situations where surgical treatment of thoracolumbar fractures involves shared decision-making with the patient, providing layman explanations can offer additional context, helping patients better prepare for surgical discussions and be more active participants in the clinical-decision making process. There may eventually be a role for ChatGPT to assess MRI reports and determine the urgency of clinical follow-up with a spine surgeon.

In contrast, there are considerable downsides for patients who may become overconfident in using ChatGPT and ultimately make poorly informed clinical decisions on their own without an expert opinion. Thus, smooth integration of LLMs, like GPT-4o, into the existing clinical infrastructure would be ideal, rather than having patients use it on their own offline and outside of their electronic health record. For example, automated layman explanations could be sent along with the original MRI report when it is released to the patient in their medical portal. In this scenario, ideally both the Radiologist and the spine surgeon would have the opportunity to proofread the ChatGPT-generated layman explanations before the reports are released to the patient, allowing patients to have an easy to understand report that has been approved by a specialized healthcare provider.

### Conclusions

While further quantitative studies are necessary, the initial insights from this study demonstrate that ChatGPT-generated layman explanations of MRI reports for thoracolumbar spine trauma are both accurate and helpful. Patient self-directed internet research often leads to clinicians having to spend extra time correcting misconceptions about their conditions. However, more structured prompting of modern LLMs, such as ChatGPT, can improve patients’ understanding of medical terminology and their conditions in an efficient and easily accessible manner. As AI tools continue to advance, surgeon oversight and evaluation will become increasingly necessary to safely integrate generative AI assistance into patient care.

## Abbreviations

AI: artificial intelligence
FKGL: Flesch-Kincaid Grade Level
FRES: Flesch Readability Ease Score
LLM: large language model
MRI: magnetic resonance imaging
PACS: Picture Archiving and Communications System

## References

[1] (2020). 21st Century Cures Act. FDA.

[2] Johnson AJ, Easterling D, Williams LS, Glover S, Frankel RM (2009). Insight from patients for radiologists: improving our reporting systems. J Am Coll Radiol.

[3] Li H, Moon JT, Iyer D (2023). Decoding radiology reports: potential application of OpenAI ChatGPT to enhance patient understanding of diagnostic reports. Clin Imaging.

[4] Jeblick K, Schachtner B, Dexl J (2024). ChatGPT makes medicine easy to swallow: an exploratory case study on simplified radiology reports. Eur Radiol.

[5] Oner FC, van Gils AP, Dhert WJ, Verbout AJ (1999). MRI findings of thoracolumbar spine fractures: a categorisation based on MRI examinations of 100 fractures. Skeletal Radiol.

[6] Dvorak MF, Öner CF, Schnake K, Dandurand C, Muijs S (2024). From radiographic evaluation to treatment decisions in neurologically intact patients with thoraco-lumbar burst fractures. Global Spine J.

[7] Ghanem D, Shu H, Bergstein V (2024). Educating patients on osteoporosis and bone health: can “ChatGPT” provide high-quality content?. Eur J Orthop Surg Traumatol.

[8] Hoang T, Liou L, Rosenberg AM (2024). An analysis of ChatGPT recommendations for the diagnosis and treatment of cervical radiculopathy. J Neurosurg Spine.

[9] Temel MH, Erden Y, Bağcıer F (2024). Information quality and readability: ChatGPT’s responses to the most common questions about spinal cord injury. World Neurosurg.

[10] Scott B (2023). Readability scoring system. Readability Formulas.

[11] Yi PH, Golden SK, Harringa JB, Kliewer MA (2019). Readability of lumbar spine MRI reports: will patients understand?. AJR Am J Roentgenol.

[12] Fan X, Zhu Q, Tu P, Joskowicz L, Chen X (2023). A review of advances in image-guided orthopedic surgery. Phys Med Biol.

[13] Rabah NM, Levin JM, Winkelman RD, Mroz TE, Steinmetz MP (2020). The association between physicians’ communication and patient-reported outcomes in spine surgery. Spine (Phila Pa 1976).

[14] Spina A, Andalib S, Flores D, Vermani R, Halaseh FF, Nelson AM (2024). Evaluation of generative language models in personalizing medical information: instrument validation study. JMIR AI.

[15] Encalada S, Gupta S, Hunt C (2025). Optimizing patient understanding of spine MRI reports using AI: a prospective single center study. Interv Pain Med.

[16] Truhn D, Weber CD, Braun BJ (2023). A pilot study on the efficacy of GPT-4 in providing orthopedic treatment recommendations from MRI reports. Sci Rep.

[17] Kuckelman IJ, Wetley K, Yi PH, Ross AB (2024). Translating musculoskeletal radiology reports into patient-friendly summaries using ChatGPT-4. Skeletal Radiol.

[18] Lyu Q, Tan J, Zapadka ME (2023). Translating radiology reports into plain language using ChatGPT and GPT-4 with prompt learning: results, limitations, and potential. Vis Comput Ind Biomed Art.

[19] Oztermeli AD (2025). Is ChatGPT a reliable tool for explaining medical terms?. Cureus.

[20] Herzog R, Elgort DR, Flanders AE, Moley PJ (2017). Variability in diagnostic error rates of 10 MRI centers performing lumbar spine MRI examinations on the same patient within a 3-week period. Spine J.

[21] Bhandari A (2024). Revolutionizing radiology with artificial intelligence. Cureus.

[22] Mazurowski MA, Buda M, Saha A, Bashir MR (2019). Deep learning in radiology: an overview of the concepts and a survey of the state of the art with focus on MRI. J Magn Reson Imaging.

[23] Wu JT, Syed A, Ahmad H (2020). AI accelerated human-in-the-loop structuring of radiology reports. AMIA Annu Symp Proc.

[24] de Almeida Prado RM, de Almeida Prado JLM, Yamada AF (2021). Spine trauma: radiological approach and new concepts. Skeletal Radiol.

[25] Navigli R, Conia S, Ross B (2023). Biases in large language models: origins, inventory, and discussion. J Data Inf Qual.

[26] Marty H, Bogenstätter Y, Franc G, Tschan F, Zimmermann H (2013). How well informed are patients when leaving the emergency department? comparing information provided and information retained. Emerg Med J.

[27] Strøm J, Bjerrum MB, Nielsen CV (2018). Anxiety and depression in spine surgery—a systematic integrative review. Spine J.

